# AI Chatbots as Sources of STD Information: A Study on Reliability and Readability

**DOI:** 10.1007/s10916-025-02178-z

**Published:** 2025-04-03

**Authors:** Hüseyin Alperen Yıldız, Emrullah Söğütdelen

**Affiliations:** Faculty of Medicine, Bolu Abant İzzet Baysal University, 14030 Bolu, Türkiye

**Keywords:** AI-generated health information, Health Comminucation, Sexually Transmitted Diseases

## Abstract

**Background:**

Artificial intelligence (AI) chatbots are increasingly used for medical inquiries, including sensitive topics like sexually transmitted diseases (STDs). However, concerns remain regarding the reliability and readability of the information they provide. This study aimed to assess the reliability and readability of AI chatbots in providing information on STDs. The key objectives were to determine (1) the reliability of STD-related information provided by AI chatbots, and (2) whether the readability of this information meets the recommended standarts for patient education materials.

**Methods:**

Eleven relevant STD-related search queries were identified using Google Trends and entered into four AI chatbots: ChatGPT, Gemini, Perplexity, and Copilot. The reliability of the responses was evaluated using established tools, including DISCERN, EQIP, JAMA, and GQS. Readability was assessed using six widely recognized metrics, such as the Flesch-Kincaid Grade Level and the Gunning Fog Index. The performance of chatbots was statistically compared in terms of reliability and readability.

**Results:**

The analysis revealed significant differences in reliability across the AI chatbots. Perplexity and Copilot consistently outperformed ChatGPT and Gemini in DISCERN and EQIP scores, suggesting that these two chatbots provided more reliable information. However, results showed that none of the chatbots achieved the 6th-grade readability standard. All the chatbots generated information that was too complex for the general public, especially for individuals with lower health literacy levels.

**Conclusion:**

While Perplexity and Copilot showed better reliability in providing STD-related information, none of the chatbots met the recommended readability benchmarks. These findings highlight the need for future improvements in both the accuracy and accessibility of AI-generated health information, ensuring it can be easily understood by a broader audience.

**Supplementary Information:**

The online version contains supplementary material available at 10.1007/s10916-025-02178-z.

## Introduction

Sexually transmitted diseases (STDs) are a sensitive and private subject, often associated with stigma and embarrassment. Many people may struggle to consult or get information from medical professionals on such matters. Instead, people increasingly tend to seek medical information online through search engines and other digital resources [1]. The rise of artificial intelligence (AI) chatbots that provide instant and easily accessible information has revolutionized the way individuals obtain health-related information [2, 3]. Currently, AI-driven tools have started to take the place of traditional search engines in conducting searches for medical-related queries [2]. This also applies to rather sensitive issues such as sexually transmitted diseases. Programs such as ChatGPT®, Gemini®, Perplexity®, and Copilot® have quickly become widely used due to their ease of use and conversational-style responses [4].

Sexually transmitted infections (STIs) remain a major global health concern. More than 1 million curable STIs are acquired every day worldwide among individuals aged 15–49 years, with the majority of cases being asymptomatic, contributing to delayed diagnoses and further transmission [5]. Despite this, limited access to healthcare services and concerns over stigma often lead individuals to seek health information online rather than consulting medical professionals. This has made AI-driven chatbots an emerging tool for disseminating health-related content, including information on STDs. This emphasizes the critical importance of providing individuals with accessible, accurate, and reliable information to help them recognize and manage these infections.

As AI chatbots become more popular, it is important to analyze the reliability and readability of the information they provide, especially for health topics that require accurate guidance. Since these AI chatbots are developing so quickly and becoming deeply ingrained in everyday life, the quality of the information they provide may seriously impact users’ perception and decision-making regarding their health.

Healthcare authorities, including the American Medical Association (AMA), the National Institutes of Health (NIH), and the United States Department of Health and Human Services (HHS), recommend that patient education materials be written at a 6th-grade reading level or below to make them accessible to a wider audience [6]. This is especially important for health topics like STDs, because understanding of prevention, treatment, and risks can directly affect public health.

Studies have highlighted variability in the quality and readability of large language model (LLM)-generated responses across medical topics, from urological malignancies to lifestyle modifications for chronic diseases [7, 8]. For instance, comparative analyses of AI chatbots across various medical conditions have revealed differences in their performance based on readability indices and adherence to clinical guidelines [9–11].

Research on AI chatbots in urology specifically has identified moderate to high-quality responses for some conditions, but also noted limitations in their ability to provide actionable, user-friendly information [7–9, 11–14]. These findings underline the need for further exploration into the specific strengths and weaknesses of LLMs, particularly in providing reliable, understandable, and comprehensive information for patient use.

This research seeks to answer two key questions: (1) How reliable is the information provided by AI chatbots regarding STDs? (2) Does the readability of the information meet the standards recommended for patient education materials? Unlike previous studies that have examined AI chatbot accuracy in broader medical topics, this study specifically focuses on STD-related queries, an area where misinformation can have serious public health consequences. Additionally, it evaluates multiple chatbot models and employs established quality assessment metrics to provide a comprehensive analysis. By addressing these questions, this study will offer insights into the potential role of AI chatbots as reliable and accessible sources of health information for sensitive topics like sexually transmitted diseases.

## Methods

The study began by identifying standardized entry terms for “sexually transmitted diseases” using the Medical Subject Headings (MeSH) database to ensure comprehensive terminology coverage. The following MeSH entry terms were identified: Disease, Sexually Transmitted; Diseases, Sexually Transmitted; Sexually Transmitted Disease; Sexually Transmitted Infections; Infection, Sexually Transmitted; Infections, Sexually Transmitted; Sexually Transmitted Infection; Transmitted Infection, Sexually; Transmitted Infections, Sexually; STIs/STI; STDs/STD; Venereal Diseases; Diseases, Venereal, Disease, Venereal; Venereal Disease.

When these MeSH entry terms were input into Google Trends, the platform provided a unified “Sexually Transmitted Diseases” topic. As Google describes it, “topics are a group of terms that share the same concept in any language,” potentially containing the most associated information [15]. While this allowed the study to capture global search trends related to STDs comprehensively, it may not capture all aspects of STD-related inquiries. This limitation is acknowledged and considered when interpreting the results. The most relevant search queries related to the topic “sexually transmitted diseases” (STDs) from the past five years (2018–2023), worldwide, were identified. Google Trends was chosen for its ability to reflect real-world search behaviors and public interest over time [16–18]. The 25 most relevant search queries were extracted, and those that were either unrelated or duplicative were removed, resulting in 11 distinct and relevant search queries (Table 1). These queries were input into each of the four AI-based programs as prompts wihout any modifications, and the responses were collected for further analysis.

**Table 1:** Google Trends data of the 25 most significant keywords queried globally for STD between 2009–2024

sexually transmitted diseases (Worldwide)	
TOP	Relevance
1. what (removed)	100
2. sex transmitted diseases (removed)	74
3. what is sexually transmitted diseases (removed)	70
4. **sexually transmitted diseases symptoms**	68
5. **what are sexually transmitted diseases**	50
6. sexually transmitted disease (removed)	42
7. disease (removed)	40
8. what are the sexually transmitted diseases (removed)	37
9. std (removed)	30
10. sexual diseases (removed)	28
11. **sexual transmitted diseases**	28
12. sexual (removed)	28
13. **gay sexually transmitted diseases symptoms**	27
14. gay	25
15. sexually transmitted infection (removed)	23
16. **sexually transmitted diseases list**	21
17. symptoms of sexually transmitted diseases (removed)	21
18. **hiv**	21
19. **sexually transmitted diseases meaning**	18
20. oral (removed)	16
21. sexually transmitted infections (removed)	16
22. **herpes**	15
23. **gonorrhea**	15
24. **chlamydia**	14
25. **types of sexually transmitted diseases**	14

ChatGPT-4o, Gemini 1.5, Perplexity Pro, and Copilot (version: 1.240.110.0) were used in our study. Before each query was entered, all browser-related data was cleared to prevent any potential bias. This step was taken to ensure that the AI models responded solely based on their internal algorithms and not on any cached data or previous interactions.

The readability of the responses was assessed using six widely recognized readability metrics, using online calculator (https://readabilityformulas.com).

- Automated Readability Index (ARI) [19]: $4.71(\frac{characters}{words})+0.5(\frac{words}{sentences})-21.43$
- Flesch Reading Ease Score (FRES) [20]: $206.835-1.015</span>(\frac{words}{sentences})-84.6(\frac{syllables}{words})$
- Gunning Fog Index (GFI) [20]: $0.4[(\frac{words}{sentences})+100(\frac{complex words}{words})]$
- Flesch-Kincaid Grade Level (FKGL) [20]: $0.39(\frac{words}{sentences})+11.8(\frac{syllables}{words})-15.59$
- Coleman-Liau Index (CL) [21]: $5.89(\frac{characters}{words})-0.3(\frac{sentences}{words})-15.8$
- Simple Measure of Gobbledygook (SMOG) [22]: $14.430\times \sqrt{polysyllables\times \frac{30}{sentences}}+3.1291$

The readability scores were compared and analyzed with the sixth-grade level of readability recommended by the AMA and NIH [6]. The accepted readability level in the FRES formula was ≥ 80.0, while it was < 6 for the other 5 formulas.

The reliability of the information provided by the AI-based programs was evaluated using the following criteria:

- **DISCERN:** Assesses the quality of health information, particularly regarding treatment choices [23]. The DISCERN handbook offers limited guidance on interpreting overall DISCERN scores, and, to the best of our knowledge, no official subdivision of these scores has been formally established. Therefore, based on the previous literature, DISCERN scores can be categorized as follows: 63 to 75 points indicate excellent quality, 51 to 62 points indicate good quality, 39 to 50 points indicate fair quality, 27 to 38 points indicate poor quality, and 16 to 26 points indicate very poor quality [24].
- **The Ensuring Quality Information for Patients (EQIP):** Evaluates the quality of patient information provided [25]. This tool assesses various elements of content, including the clarity of information and the quality of the writing. It consists of 20 questions, each of which can be answered with “yes,” “partly,” “no,” or “does not apply.” To calculate the score, the number of “yes” responses is multiplied by 1, “partly” by 0.5, and “no” by 0. The sum of these values is divided by 20, and the number of “does not apply” responses is subtracted from this total. The final value is then multiplied by 100, yielding the EQIP score as a percentage [25]. For the study, we used the overall average EQIP score to categorize each resource into specific quality categories. The classification was based on score ranges as recommended in the original EQIP development document. Answers scoring between 76 and 100% were classified as “well written” and of excellent quality; those between 51 and 75% were considered “good quality with minor issues”; scores between 26 and 50% indicated “serious quality issues,” and those between 0 and 25% were labeled as having “severe quality problems.” [26].
- **Global Quality Scale (GQS):** Provides an overall quality score for health-related content. The GQS is a five-point Likert scale based on the quality of information, the flow of information found online, and ease of use, with 1 point very bad, 2 points bad, 3 points fair, 4 points good and 5 points excellent quality.
- **JAMA Benchmark Criteria:** Measures the adherence to standards of authorship, references and sources, indication of date, and ownership [27]. Each factor is given a score between 0 and 1, resulting in a final score that ranges from 0 to 4.

These evaluations were conducted independently by two authors, HAY and ES. Both evaluators have over 10 years of clinical experience in the field of urology. Interrater agreement was assessed to ensure consistency in scoring. In cases of disagreement, the senior urologist, UÜ, conducted the assessment, and his evaluation was accepted as the final score.

### Statistical Analysis

#### Reliability Analysis

Descriptive statistics, including means and standard deviations, were calculated for the DISCERN, EQIP, JAMA, and GQS scores across all four programs. To determine whether the variances were homogeneous, Levene's test was applied. The Kruskal–Wallis test was employed to compare the reliability scores across the programs. Interrater agreement between the two evaluators was assessed using Cohen’s Kappa coefficient to measure the consistency of scoring. Kappa results interpreted as follows; values ≤ 0 as indicating no agreement and 0.01–0.20 as poor, 0.21–0.40 as fair, 0.41–0.60 as moderate, 0.61–0.80 as substantial, and 0.81–1.00 as almost perfect agreement [28].

#### Readability Analysis

The readability scores of the responses from each program were calculated using the aforementioned readability metrics. Due to the nature of the data, the Kruskal–Wallis test was used to compare the readability scores across the four programs, allowing for the identification of statistically significant differences without assuming a normal distribution.

#### Comparison with 6th-Grade Benchmarks

To assess the suitability of the readability scores for a 6th-grade audience, the Wilcoxon signed-rank test was employed. This test compared the AI-generated readability scores against the established 6th-grade reference values to determine if there were any statistically significant deviations.

P-values were calculated for all statistical tests, with a threshold of *p* < 0.05 considered indicative of statistically significant differences. All statistical analyses were conducted using SPSS Version 26.0. (Armonk, NY: IBM Corp.).

## Results

### Reliability Analysis

The reliability of responses was assessed using the DISCERN, EQIP, JAMA, and GQS scoring systems. Table 2 summarizes the descriptive statistics (mean ± standard deviation) for each program across the four reliability indices. There was substantial agreement between reviewers for DISCERN and EQIP, almost perfect agreement for GQS, and excellent agreement for JAMA (75.0%, kappa = 0.69; 84.1%, kappa = 0.79; 90.9%, kappa = 0.89; 100%, kappa = 1.00, respectively).

**Table 2:** Reliability Scores Across AI Chatbots

	DISCERN (mean ± SD)	EQIP (mean ± SD)	JAMA (mean ± SD)	GQS (mean ± SD)
ChatGPT	33.27 ± 6.65	52.36 ± 5.54	0.0 ± 0.0	3.36 ± 0.67
Gemini	38.09 ± 3.05	52.45 ± 3.78	1.0 ± 0.0	3.45 ± 0.52
Perplexity	42.27 ± 2.49	59.55 ± 4.99	1.0 ± 0.0	3.45 ± 0.52
Copilot	41.73 ± 2.2	57.91 ± 4.61	1.0 ± 0.0	3.45 ± 0.52
p	< 0.001	0.0048	< 0.001	0.994

The Kruskal–Wallis test revealed significant differences across programs for DISCERN (*p* < 0.001), EQIP (*p* = 0.0048), and JAMA (*p* < 0.001) scores. No significant differences were observed for GQS (*p* = 0.994).

**Fig. 1:**
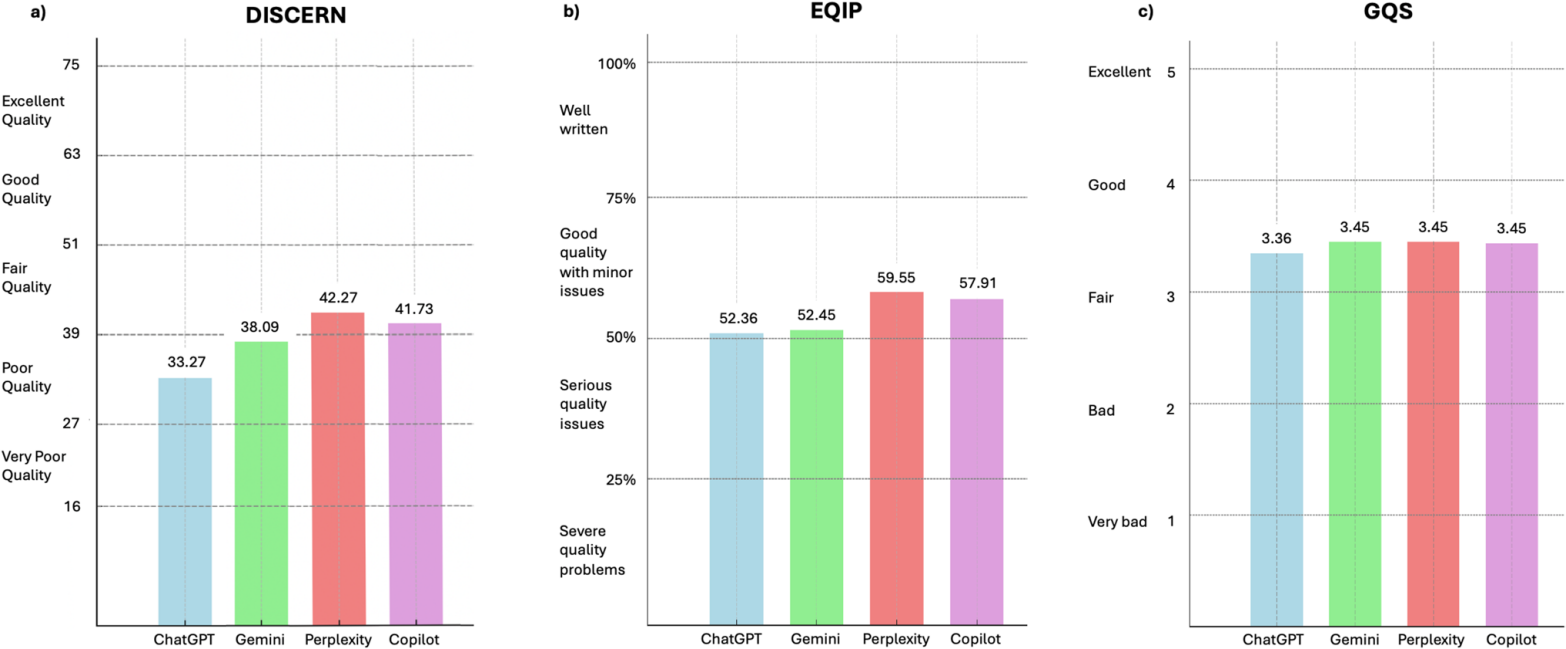
Mean reliability scores of the chatbots based on (**a**) DISCERN, (**b**) EQIP and (**c**)GQS indexes

Based on the DISCERN scores, the quality of information provided by ChatGPT and Gemini was classified as poor, while Perplexity and Copilot were rated as providing good quality information (Fig. 1). This highlights a significant difference in the reliability of responses across the chatbots, with ChatGPT and Gemini providing lower-quality information due to issues with transparency and sourcing; Perplexity and Copilot had the highest scores, indicating that these chatbots provided more structured and reliable information.

For the EQIP scores, all four chatbots were rated as “good quality with minor issues”, indicating that the overall structure and clarity of the information were satisfactory, though some improvements could be made in areas such as readability and additional information sources (Fig. 1).

Finally, according to the GQS scores, all chatbots were evaluated as providing fair information (Fig. 1). This suggests that while the chatbots are generally adequate in providing health information, there is still room for improvement in terms of how effectively they communicate critical health details to users.

Pairwise comparisons using the Mann–Whitney U test showed that Perplexity and Copilot consistently scored higher on DISCERN and EQIP compared to ChatGPT and Gemini. (Table 3).

**Table 3:** Mann–Whitney U Test Results for Reliability Scores (p-values)

Pairwise comparison	DISCERN	EQIP	JAMA	GQS
ChatGPT vs Gemini	0.0845	1.0	0.0	0.8528
ChatGPT vs Perplexity	0.0014	0.0066	0.0	0.8528
ChatGPT vs Copilot	0.0013	0.0553	0.0	0.8528
Gemini vs Perplexity	0.0034	0.0024	1.0	1.0
Gemini vs Copilot	0.0055	0.045	1.0	1.0
Perplexity vs Copilot	0.643	0.4371	1.0	1.0

These results indicate that Perplexity and Copilot provided more reliable information (as measured by DISCERN and EQIP), particularly compared to ChatGPT. ChatGPT’s lower JAMA score suggests that it adhered more closely to professional medical publishing standards, such as authorship and reference use.

### Readability Analysis

Readability was evaluated using six widely recognized indices: ARI, FRES, GFI, FKGL, CL, and SMOG Index. The average readability scores for each chatbot are shown in Table 4.

**Table 4:** Readability Scores Across AI Chatbots

Program	ARI (mean ± SD)	FRES (mean ± SD)	GFI (mean ± SD)	FKGL (mean ± SD)	CL (mean ± SD)	SMOG (mean ± SD)
ChatGPT	11.13 ± 2.21	41.55 ± 9.06	13.46 ± 2.17	10.34 ± 1.87	13.37 ± 1.95	9.34 ± 1.8
Gemini	8.75 ± 1.63	48.27 ± 6.8	11.19 ± 1.07	8.43 ± 1.04	12.02 ± 1.72	7.51 ± 0.82
Perplexity	10.42 ± 1.35	43.27 ± 7.11	13.2 ± 1.6	10.07 ± 1.18	13.26 ± 1.28	9.48 ± 1.03
Copilot	10.69 ± 2.13	43.82 ± 9.94	13.41 ± 2.35	10.04 ± 1.68	13.11 ± 1.98	9.62 ± 1.68
6th grade level score	6	80–90	6	6	6	6

^*^ARI: Automated Readability Index, FRES: Flesch Reading Ease Score, GFI: Gunning Fog Index, FKGL: Flesch-Kincaid Grade Level, CL: Coleman-Liau Index, SMOG: Simple Measure of Gobbledygook, SD: Standart Deviation

**Fig. 2:**
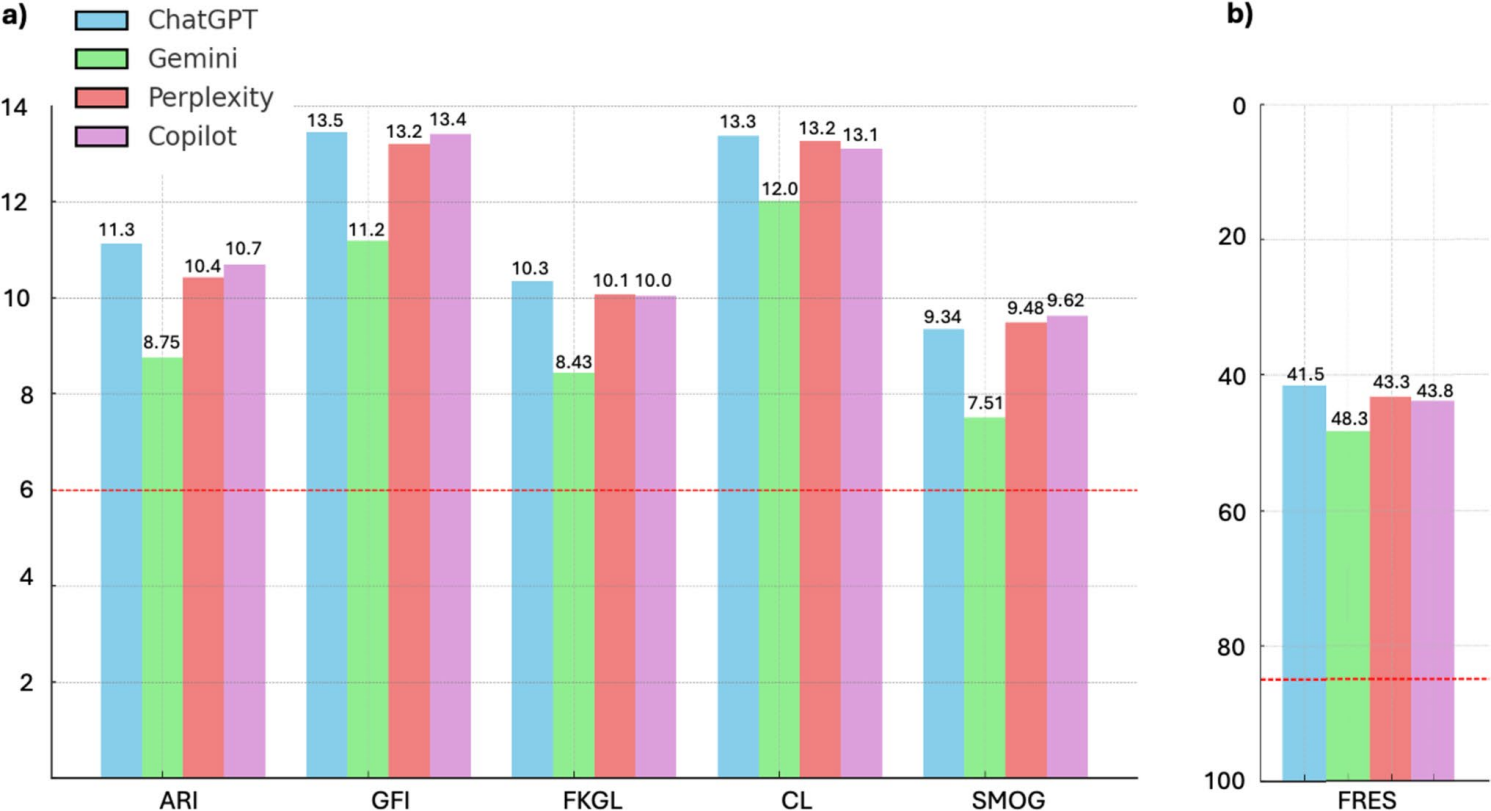
Mean readability scores of the chatbots based on (**a**) ARI, GFI, FKGL, CL, SMOG and, (**b**) FRES indexes. Red line indicates the 6th-grade level which is the highest recommended reading level for patient education materials. ARI: Automated readability index, GFI: Gunning Fog index, FKGL: Flesch-Kincaid grade level, CL: Coleman-Liau index, SMOG: Simple measure of gobbledygook, FRES: Flesch reading ease score

None of the chatbots met the 6th-grade readability benchmark across all indices. The FRES were significantly lower than the recommended 80–90 range for the 6th-grade level (Fig. 2). Similarly, the GFI and FKGL suggest that the text complexity exceeded what is appropriate for a general audience (Fig. 2).

The Wilcoxon signed-rank test was used to compare each chatbot’s readability scores against the 6th-grade benchmarks. None of the chatbots’ responses consistently met the readability standards across all indices (*p* = 0.125).

These results indicate that, despite their reliability, the responses from all four chatbots were too complex to be easily understood by the general public, especially at the recommended 6th-grade reading level.

## Discussion

AI chatbots are increasingly being used for sensitive health-related queries, it has become imperative to understand the quality of information passed through such chatbots and, importantly, whether it can be comprehended by the general public. The results of this study and prior research indicate that they have incosistent performance in delivering high-quality, easily understandable information.

### Reliability of AI-Generated Health Information

This study utilized DISCERN, EQIP, GQS, and JAMA indices to evaluate the reliability and quality of chatbot-generated health information. Each index was selected for its specific advantages and ability to assess different aspects of information quality and reliability. DISCERN and EQIP are well-established tools designed to evaluate the reliability and quality of written health materials. A prior study comparing these two scales found that both exhibit high reliability; however, DISCERN demonstrated superior inter-rater agreement, making it particularly effective for consistent evaluations across different reviewers [29]. DISCERN focuses on criteria such as the clarity, balance, and comprehensiveness of health-related information, while EQIP emphasizes more detailed content-specific evaluation, particularly in clinical settings. In contrast, GQS is a simpler and more subjective scoring system aimed at assessing the overall quality of information. While it lacks the depth and structure of DISCERN and EQIP, it provides a valuable perspective by considering user perception and the general utility of the material. JAMA, on the other hand, evaluates credibility based on authorship, attribution, disclosure, and currency.

Despite their utility, these indices were originally developed for evaluating static, written materials and may not be perfectly suited for assessing chatbot-generated responses. Chatbots, while producing written responses, differ from traditional written materials in their conversational nature and dynamic content generation. Nevertheless, studies have shown correlations between these indices, suggesting that their combined use offers a comprehensive evaluation of both reliability and quality from multiple perspectives [30]. By employing these diverse scoring systems, we aimed to provide a more nuanced understanding of chatbot performance.

Perplexity and Copilot outperformed ChatGPT and Gemini across several reliability metrics, particularly DISCERN and EQIP. This finding underlines the importance of source attribution in perceived reliability. Perplexity and Copilot were able to achieve higher reliability scores by consistently citing sources or providing clear references. In contrast, ChatGPT, which lacks verifiable sources, scored significantly lower on both the DISCERN and JAMA indices [31]. The lower scores for ChatGPT and Gemini may reflect their more generalized approach, where the emphasis on sourcing and authorship is not a prioritized. This can make it difficult to assess the credibility of the information, reducing trust in medical recommendations. Source transparency is a key determinant of perceived credibility in medical information. Users increasingly rely on AI chatbots for health-related queries, and when chatbots fail to cite reliable sources, they risk disseminating incomplete or potentially misleading information. This is particularly concerning in sensitive health topics such as STDs, where misinformation can lead to harmful consequences, including delays in seeking medical care or the spread of inaccurate prevention strategies.

### Readability Challenges

Readability was another major focus of this study. Multiple readability indices were employed to comprehensively assess the complexity of chatbot-generated health information. While indices like Flesch Reading Ease and Flesch-Kincaid are widely used in the literature, prior research suggests that the SMOG formula may be better suited for evaluating healthcare-related materials [32]. This is because SMOG has been validated against 100% comprehension, is based on more recent criteria for determining reading grade levels [32]. By including all these indices, our analysis aimed to capture a holistic view of readability across different metrics. The results were consistent across all indices, indicating that the readability levels of all chatbot responses exceeded the recommended 6th-grade threshold, reinforcing the challenge these systems face in delivering easily understandable information to users with lower health literacy. This approach also allowed us to identify potential areas for improvement in simplifying the language and structure of AI-generated health content. For example, a response from ChatGPT regarding chlamydia treatment included technical terminology such as “macrolide antibiotic” and “bacterial protein synthesis inhibition at the ribosomal level,” which may be difficult for lay users to interpret. In contrast, a more patient-friendly phrasing would simplify the explanation by stating that chlamydia can be treated with antibiotics that stop bacterial growth.

This represents that in the future, AI-powered chatbots will be able to provide a great deal of information, but the level of difficulty of such responses could be not easy to interpret for users who have low levels of literacy. Given the importance of health literacy, it is of concern that information provided by these chatbots may not equally be accessible to all users. In sensitive topics where clear and easy-to-understand information is crucial, this represents a potential limitation with current AI chatbot technology.

### Review of Previous Literature

Previous studies examining AI chatbots for medical contexts have similarly identified issues regarding source transparency and the readability of information provided. For example, in a study that compared the performance of five different chatbots on penile prosthesis-related questions, ChatGPT had lower reliability because there were no source citations, resulting in lower DISCERN scores [33]. Similarly in our study, ChatGPT and Gemini were classified as poor quality based on their DISCERN scores, while Perplexity and Copilot performed significantly better. This suggests that source citation transparency is very critical to creating trust in medical information generated on chat interfaces.

Likewise, a research evaluating the quality of chatbot-generated responses in urological malignancies found that while chatbots like Perplexity and Copilot offer reliable, accurate information, their readability remains an issue, with responses often written at a high reading level, making them inaccessible to patients with lower health literacy [7].

Another study evaluating kidney stone patient information materials also found that although AI chatbots improve accessibility to medical information, they often fail to meet readability guidelines, where their content mostly being too complex for the average patient [14].

A study evaluating AI chatbot responses to erectile dysfunction queries found significant differences in quality and readability between chatbots [13]. ChatGPT, Copilot, Bard, and others demonstrated variable performance across metrics like DISCERN, EQIP, and readability scores [13]. Another study examining premature ejaculation information from ChatGPT further support this, also report similarly low scores on EQIP and DISCERN, with the language used being complex [9].

The poor readability of chatbot-generated medical information is consistently highlighted in these studies. Responses related to erectile dysfunction-related topics, such as urological health and premature ejaculation, did not meet the recommended readability grade level in any of the studied chatbots [9, 12, 13]. This was consistent with the findings of the present study.

### Limitations

There are several limitations to this study that should be acknowledged. First, the analysis was based on a small sample of AI chatbot responses, specifically limited to sexually transmitted diseases. While this focus allowed for a detailed evaluation in one subject, the results may not be fully generalizable to other medical topics.

Another limitation is the dataset size and the potential for inherent biases in chatbot responses. The study relied on a limited number of queries, which may not fully capture the breadth of user inquiries regarding STDs. AI chatbots generate responses based on their training data, which inherently includes biases from the sources they were trained on. These biases can influence the accuracy, completeness, and framing of the information provided. Additionally, variations in chatbot responses due to different phrasing of queries or context-specific nuances could affect reliability assessments.

The use of Google Trends in this study comes with certain limitations. One key limitation is the lack of detailed demographic information, such as age and sex, as well as the inability to determine the exact sample size of the data. While previous research has demonstrated that web-based data can provide valuable and valid insights into behavior and often correlate with actual data, the reliability of online queries may be compromised in regions with low internet penetration [16–18, 34]. However, since our study focuses specifically on internet users, we consider these findings suitable for our objectives. Furthermore, as we conducted a worldwide analysis, the variation in questions asked about the topic across different regions, linguistic diversity and cultural nuances could influence the results [35, 36]. Despite these constraints, Google Trends remains a useful tool for examining global patterns in online health information searches.

The study relied on established readability and reliability metrics, which may not fully capture the nuances of AI-generated content. While DISCERN or Flesch Reading Ease gives important insight to the quality and accessibility of the information, neither of them take into account subjective factors like user trust or conversational tone generated by chatbots. Future research should explore modern evaluation frameworks, such as machine learning-based quality assessment models or real-time user engagement metrics, to better assess the reliability, readability, and practical utility of AI-generated health information.

Lastly, the AI chatbot models are constantly evolving, they get updated regularly. The versions of the model evaluated in this study may differ from future versions. This could impact the reproducibility of these results over time. Regular evaluations will be needed to see how the performance of these AI systems changes as they are updated.

### Practical Implications

The findings of this study have important implications for the development and implementation of AI chatbots in healthcare. Among the evaluated chatbots, Perplexity and Copilot emerged as the most reliable options, demonstrating higher DISCERN and EQIP scores compared to ChatGPT and Gemini. Their ability to provide clearer, more structured responses with some degree of source attribution makes them preferable for users seeking accurate medical information. However, even these chatbots did not achieve an “excellent” reliability rating, indicating the need for further improvements in the transparency and quality of AI-generated content.

The inconsistent reliability and poor readability of chatbot responses highlight the need for improvements in both areas. For healthcare providers and developers, this means prioritizing the inclusion of transparent citation mechanisms and simplifying the language used in responses. Ensuring that chatbot-generated content is accessible to users with lower health literacy is essential for equitable access to health information.

From a public health perspective, these findings underscore the importance of responsible AI deployment in medical communication. Healthcare professionals should be aware of chatbot limitations and guide patients toward more reliable information sources when necessary. AI chatbots, while promising, should be regarded as supplementary tools rather than replacements for professional medical consultation.

### Future Directions

Future research should focus on developing new evaluation frameworks tailored to the unique characteristics of chatbot-generated content. These frameworks should account for the conversational nature and dynamic generation of responses, which differ from traditional written materials. Additionally, further studies are needed to explore how chatbot updates impact performance over time and to identify strategies for improving both reliability and readability. Expanding the scope of analysis to include diverse medical topics will also help generalize findings and enhance the practical utility of chatbots in healthcare.

Moreover, the inconsistent performance across chatbots suggests that current AI models vary significantly in how they retrieve and present medical knowledge. This highlights the need for standardized guidelines in AI-driven health communication, ensuring that chatbots provide evidence-based, clearly referenced, and easily verifiable content. Developers should prioritize integrating structured citation mechanisms and improving fact-verification algorithms to enhance the reliability of chatbot-generated medical advice. To enhance the reliability and accessibility of AI-generated health information, future research should explore ways to integrate domain-specific data into chatbot training models. Additionally, refining readability algorithms by simplifying sentence structures and reducing technical jargon could significantly improve user comprehension.

To ensure continuous assessment of AI chatbot reliability and readability, future studies should consider a longitudinal evaluation strategy. Given the frequent updates and refinements of chatbot models, periodic reassessments are necessary to track changes in their performance over time. A structured framework for ongoing evaluations would provide valuable insights into how modifications affect chatbot-generated health information. Such an approach could also aid in identifying trends and improvements in chatbot reliability and readability.

## Conclusion

This study underlines the need for continuous evaluation of AI chatbots in the healthcare domain. While chatbots like Perplexity and Copilot demonstrate stronger performance in terms of reliability, significant work remains to improve the readability of the information they provide. Moving forward, it is essential that AI chatbot developers work to simplify language, improve source transparency, and ensure that the information provided meets both the reliability and readability needs of diverse users. Achieving this balance will be crucial for the successful integration of AI chatbots into healthcare communication.

## Supplementary Information

Below is the link to the electronic supplementary material.

Supplementary file1 (DOCX 26 KB)

## Data Availability

No datasets were generated or analysed during the current study.

## References

[1] Sun, J., Zhang, S., Hou, M., Sun, Q., Cao, F., Zhang, Z., Tang, G., Wang, X., Geng., L., Cui, L., and Chen, Z.-J., Who can help me? Understanding the antecedent and consequence of medical information seeking behavior in the era of bigdata. *Front Public Health*. 11:1192405, 2023. 10.3389/fpubh.2023.1192405 10.3389/fpubh.2023.1192405 PMC10544578 37790712

[2] Noorbakhsh-Sabet N, Zand R, Zhang Y, Abedi V (2019) Artificial Intelligence Transforms the Future of Health Care. Am J Med 132:795–801. 10.1016/j.amjmed.2019.01.017 30710543 10.1016/j.amjmed.2019.01.017 PMC6669105

[3] Swire-Thompson B, Lazer D (2020) Public Health and Online Misinformation: Challenges and Recommendations. Annu Rev Public Health 41:433–451. 10.1146/annurev-publhealth-040119-094127 31874069 10.1146/annurev-publhealth-040119-094127

[4] Radfort, A., Narasimhan, K., Salimans, T., and Sutskever, I., (OpenAI Transformer): Improving language understanding by Generative Pre-Training. OpenAI. (2018). https://cdn.openai.com/research-covers/language-unsupervised/language_understanding_paper.pdf. Accessed 20 Sep 2024.

[5] World Health Organization., Global and regional STI estimates. WHO. 2024. https://www.who.int/data/gho/data/themes/topics/global-and-regional-sti-estimates. Accesed 20 Sept 2024.

[6] Weiss BD (2007) Health literacy and patient safety: Help patients understand (manual for clinicians, second edition). American Medical Association Foundation and American Medical Association

[7] Musheyev D, Pan A, Loeb S, Kabarriti AE (2024) How Well Do Artificial Intelligence Chatbots Respond to the Top Search Queries About Urological Malignancies? Eur Urol 85:13–16. 10.1016/j.eururo.2023.07.004 37567827 10.1016/j.eururo.2023.07.004

[8] Wang, J., and Yun, X., Accuracy and readability of kidney stone patient information materials generated by a large language model compared to official urologic organizations. Urol. 189:e12, 2024. 10.1016/j.urology.2024.03.029 10.1016/j.urology.2024.03.029 38653389

[9] Şahin., M. F., Keleş, A., Özcan, R., Doğan, Ç., Topkaç, E. C., Akgül, M., and Yazıci, C. M., Evaluation of information accuracy and clarity: ChatGPT responses to the most frequently asked questions about premature ejaculation. *Sex Med.* 12:qfae036, 2024. 10.1093/sexmed/qfae036 10.1093/sexmed/qfae036 PMC11144523 38832125

[10] Talyshinskii, A., Naik, N., Hameed, B. M. Z., Juliebø-Jones, P., and Somani, B. K., Potential of AI-driven chatbots in urology: Revolutionizing patient care through artificial intelligence. *Curr Urol Rep*. 25:9–18, 2024. 10.1007/s11934-023-01184-3 10.1007/s11934-023-01184-3 PMC10787686 37723300

[11] Whiles, B. B., Bird, V. G., Canales, B. K., DiBianco, J. M., and Terry, R. S., Caution! AI bot has entered the patient chat: ChatGPT has limitations in providing accurate urologic healthcare advice. Urol. 180:278–284, 2023. 10.1016/j.urology.2023.07.010 10.1016/j.urology.2023.07.010 37467806

[12] Szczesniewski JJ, Tellez Fouz C, Ramos Alba A, Diaz Goizueta FJ, García Tello A, Llanes González L (2023) ChatGPT and most frequent urological diseases: analysing the quality of information and potential risks for patients. World J Urol 41:3149–3153. 10.1007/s00345-023-04563-0 37632558 10.1007/s00345-023-04563-0

[13] Şahin MF, Ateş H, Keleş A, Özcan R, Doğan Ç, Akgül M, Yazıcı CM (2024) Responses of Five Different Artificial Intelligence Chatbots to the Top Searched Queries About Erectile Dysfunction: A Comparative Analysis. J Med Syst 48:38. 10.1007/s10916-024-02056-0 38568432 10.1007/s10916-024-02056-0 PMC10990980

[14] Kim, S. H., Tae, J. H., Chang, I. H., Kim, T.-H., Myung, S. C., Nguyen, T. T., Choi, J., Kim, J. H., Kim, J. W., Lee, Y. S., and Choi, S. Y., Changes in patient perceptions regarding ChatGPT-written explanations on lifestyle modifications for preventing urolithiasis recurrence. *Digit Health*. 9:20552076231203940, 2023. 10.1177/20552076231203940 10.1177/20552076231203940 PMC10540569 37780059

[15] Mavragani, A., and Ochoa, G., Google trends in infodemiology and infoveillance: Methodology framework. *JMIR Public Health Surveill.* 5:e13439, 2019. 10.2196/13439 10.2196/13439 PMC6660120 31144671

[16] Scharkow, M., and Vogelgesang, J., Measuring the public agenda using search engine queries. Int J Public Opin Res. 23:104–113, 2011. 10.1093/ijpor/edq048

[17] Xu, C., Wang, Y., Yang, H., Hou, J., Sun, L., Zhang, X., Cao, X., Hou, Y., Wang, L., Cai, Q., and Wang, Y. Association between cancer incidence and mortality in web-based data in China: Infodemiology study. *J Med Internet Res*. 21:e10677, 2019. 10.2196/10677 10.2196/10677 PMC6371071 30694203

[18] Watad, A., Watad, S., Mahroum, N., Sharif, K., Amital, H., Bragazzi, N. L., and Adawi, M. Forecasting the west nile virus in the United States: An extensive novel data streams-based time series analysis and structural equation modeling of related digital searching behavior. *JMIR Public Health Surveill.* 5:e9176, 2019. 10.2196/publichealth.9176 10.2196/publichealth.9176 PMC6416538 30601755

[19] Smith, E. A., and Senter, R. J., Automated readability index. *AMRL-TR Aerospace Medical Research Laboratories*. 1–14, 1967. https://apps.dtic.mil/sti/pdfs/AD0667273.pdf 5302480

[20] Kincaid, P. J., Derivation of new readability formulas. Chief of Naval Technical Training, Naval Air Station Memphis. 1975.

[21] Coleman, M., and Liau, T. L., A computer readability formula designed for machine scoring. *J Appl Psychol*. 60:283–284, 1975. 10.1037/h0076540

[22] Hedman, A. S., Using the SMOG formula to revise a health-related document. *Am J Health Educ.* 39:61–64, 2008. 10.1080/19325037.2008.10599016

[23] Charnock D, Shepperd S, Needham G, Gann R (1999) DISCERN: an instrument for judging the quality of written consumer health information on treatment choices. J Epidemiol Community Health (1978) 53:105–111. 10.1136/jech.53.2.105 10.1136/jech.53.2.105 PMC1756830 10396471

[24] Weil AG, Bojanowski MW, Jamart J, Gustin T, Lévêque M (2014) Evaluation of the Quality of Information on the Internet Available to Patients Undergoing Cervical Spine Surgery. World Neurosurg 82:e31–e39. 10.1016/j.wneu.2012.11.003 23142585 10.1016/j.wneu.2012.11.003

[25] Moult B, Franck LS, Brady H (2004) Ensuring Quality Information for Patients: development and preliminary validation of a new instrument to improve the quality of written health care information. Health Expectations 7:165–175. 10.1111/j.1369-7625.2004.00273.x 15117391 10.1111/j.1369-7625.2004.00273.x PMC5060233

[26] Hain T (2002) Improving the quality of health information: the contribution of C‐H‐i‐Q. Health Expectations 5:270–273. 10.1046/j.1369-6513.2002.00189.x 12199665 10.1046/j.1369-6513.2002.00189.x PMC5060154

[27] Silberg, W. M., Lundberg, G. D., and Musacchio, R. A., Assessing, controlling, and assuring the quality of medical information on the internet: Caveant Lector et Viewor - Let the reader and viewer beware. *JAMA*. 277:1244–1245, 1997. 10.1001/jama.1997.03540390074039 9103351

[28] Cohen J (1960) A Coefficient of Agreement for Nominal Scales. Educ Psychol Meas 20:37–46. 10.1177/001316446002000104

[29] McCool, M. E., Wahl, J., Schlecht, I., and Apfelbacher, C., Evaluating written patient information for eczema in German: Comparing the reliability of two instruments, DISCERN and EQIP. *PLoS One.* 10:e0139895, 2015. 10.1371/journal.pone.0139895 10.1371/journal.pone.0139895 PMC4595422 26440612

[30] Genç, M., YouTube as a source of patient information on positron emission tomography. *J Health Sci Med*. 6:597–603, 2023. 10.32322/jhsm.1245143

[31] Branum, C., and Schiavenato, M., Can ChatGPT accurately answer a PICOT question? Assessing AI response to a clinical question. *Nurse Educ*. 48:231–233, 2023. 10.1097/NNE.0000000000001436 10.1097/NNE.0000000000001436 37130197

[32] Wang, L. W., Miller, M. J., Schmitt, M. R., and Wen, F. K., Assessing readability formula differences with written health information materials: Application, results, and recommendations. *Res Social Adm Pharm*. 9:503–516, 2013. 10.1016/j.sapharm.2012.05.009 10.1016/j.sapharm.2012.05.009 22835706

[33] Shayegh NA, Byer D, Griffiths Y, Coleman PW, Deane LA, Tonkin J (2024) Assessing artificial intelligence responses to common patient questions regarding inflatable penile prostheses using a publicly available natural language processing tool (ChatGPT). Can J Urol 31:11880–11885 38912940

[34] Mavragani, A., and Tsagarakis, K. P., Predicting referendum results in the Big Data Era. *J Big Data*. 6:3, 2019.

[35] Rovetta, A., Reliability of google trends: Analysis of the limits and potential of web infoveillance during COVID-19 pandemic and for future research. *Front Res Metr Anal*. 6:670226, 2021. 10.3389/frma.2021.670226 10.3389/frma.2021.670226 PMC8186442 34113751

[36] Alibudbud, R., Google trends for health research: Its advantages, application, methodological considerations, and limitations in psychiatric and mental health infodemiology. *Front Big Data*. 6:1132764, 2023. 10.3389/fdata.2023.1132764 10.3389/fdata.2023.1132764 PMC10083382 37050919

