# Supplementary material for: AI Chatbots as Sources of STD Information: A Study on Reliability and Readability
Source: J Med Syst. 2025 Apr 3;49(1):43. doi: 10.1007/s10916-025-02178-z (PMC11968469; doi:10.1007/s10916-025-02178-z)
Supplement: Supplementary file 1 — Supplementary file1 (DOCX 26 KB) [file 10916_2025_2178_MOESM1_ESM.docx]

**CHATGPT prompt 1: sexually transmitted diseases symptoms**

**CHATGPT answer 1:**

Sexually transmitted diseases (STDs), also known as sexually transmitted infections (STIs), can cause a variety of symptoms. However, some STDs can be asymptomatic, meaning that an infected person may not show any signs or symptoms. Here's a general overview of common symptoms associated with various STDs:

**1. Chlamydia**

- Often asymptomatic, especially in women.
- Painful urination.
- Abnormal vaginal or penile discharge.
- Pain during intercourse (for women).
- Testicular pain (for men).

**2. Gonorrhea**

- Painful urination.
- Abnormal discharge from the penis or vagina.
- Testicular pain (for men).
- Vaginal bleeding between periods (for women).
- Painful bowel movements and anal itching if rectally infected.

**3. Syphilis**

- **Primary stage**: A single sore or multiple sores at the infection site (usually painless).
- **Secondary stage**: Skin rashes, mucous membrane lesions, fever, swollen lymph nodes.
- **Latent stage**: No symptoms.
- **Tertiary stage**: Severe medical problems affecting the heart, brain, and other organs.

**4. Genital Herpes (HSV-2 and HSV-1)**

- Painful blisters or sores on the genital area, anus, or mouth.
- Itching or tingling sensation before the sores appear.
- Flu-like symptoms during the initial outbreak (fever, body aches).

**5. Human Papillomavirus (HPV)**

- Often asymptomatic.
- Genital warts: Small, flesh-colored or grey swellings in the genital area.
- Some strains can lead to cervical cancer and other cancers.

**6. HIV/AIDS**

- Early (acute) HIV infection may cause flu-like symptoms: fever, sore throat, fatigue.
- Persistent swollen lymph nodes.
- Weight loss, recurrent fever, and night sweats.
- Advanced stages can lead to opportunistic infections and severe immune system damage.

**7. Trichomoniasis**

- Often asymptomatic, particularly in men.
- Itching or irritation inside the penis, burning after urination or ejaculation (for men).
- Vaginal discharge (greenish, yellowish, or frothy), strong vaginal odor, itching (for women).

**8. Hepatitis B**

- Often asymptomatic in the early stages.
- Flu-like symptoms: fatigue, nausea, vomiting, abdominal pain.
- Yellowing of the skin and eyes (jaundice), dark urine, and pale stool.

**9. Mycoplasma Genitalium**

- Often asymptomatic.
- Painful urination.
- Abnormal discharge from the genital area.
- Pelvic pain in women.

**10. Pubic Lice (Crabs)**

- Intense itching in the genital area.
- Visible lice or eggs in pubic hair.
- Blue or red spots on the skin from lice bites.

**11. Scabies**

- Intense itching, especially at night.
- Rash with small red bumps or blisters.

**Important Notes:**

- **Asymptomatic Infections:** Many STDs can be present without symptoms. This makes regular testing important, especially if you are sexually active with multiple partners.
- **Complications:** Untreated STDs can lead to serious health problems, including infertility, chronic pain, and increased risk of HIV.
- **Prevention:** Use condoms, get vaccinated (for HPV and hepatitis B), and have regular medical check-ups.

If you or someone you know is experiencing symptoms or might have been exposed to an STD, it's crucial to seek medical advice and testing promptly.

**Gemini prompt 1: sexually transmitted diseases symptoms**

**Gemini answer 1:**

**Disclaimer:** This information is intended for general knowledge and informational purposes only, and does not constitute medical advice. If you suspect you may have an STD, please consult with a healthcare professional for proper diagnosis and treatment.

**STDs** are infections spread primarily through sexual contact. Symptoms can vary widely, and sometimes there may be no noticeable symptoms at all. This is why regular testing is crucial.

**Common STDs and Potential Symptoms**

- **Chlamydia:**
  - Often no symptoms
  - Burning sensation when urinating
  - Abnormal discharge from the penis or vagina
  - Painful intercourse
  - Lower abdominal pain
- **Gonorrhea:**
  - Often no symptoms
  - Burning sensation when urinating
  - Abnormal discharge from the penis or vagina
  - Painful intercourse
  - Rectal pain or bleeding
- **Syphilis:**
  - Sores on the genitals, mouth, or rectum
  - Rash
  - Hair loss
  - Fatigue
  - Fever
  - Weight loss
  - Sore throat
- **Genital Herpes:**
  - Painful blisters or sores on the genitals
  - Flu-like symptoms (fever, body aches)
  - Tender lymph nodes
- **Human Papillomavirus (HPV):**
  - Often no symptoms
  - Genital warts
  - Can lead to cervical cancer in women
- **Trichomoniasis:**
  - Itching, burning, or redness of the genitals
  - Discomfort when urinating
  - Foul-smelling vaginal discharge
  - Pain during intercourse
- **HIV/AIDS:**
  - Flu-like symptoms (fever, fatigue, sore throat)
  - Swollen lymph nodes
  - Weight loss
  - Persistent diarrhea
  - Skin rash

**When to Seek Medical Attention**

If you experience any symptoms of an STD, it's essential to consult with a healthcare provider for proper diagnosis and treatment. Early detection and treatment can prevent complications.

**Remember:** Many STDs can be treated effectively with antibiotics or other medications.

**Prevention:**

- Consistent and correct use of condoms
- Getting vaccinated against HPV
- Regular testing for STDs

Would you like to know more about a specific STD or prevention methods?

**Sources:**

<https://www.verywellhealth.com/how-long-before-std-symptoms-appear-3133026>

**Perplexity prompt 1: sexually transmitted diseases symptoms**

**Perplexity answer 1:**

The symptoms of sexually transmitted diseases (STDs) can vary depending on the specific infection, but some common signs include:

General Symptoms

- Sores, bumps, or rashes on or around the genitals, anus, buttocks, thighs, or mouth
- Painful or burning urination
- Unusual discharge from the penis or vagina
- Itchiness in or around the vagina
- Unexpected periods or bleeding after sexual activity

However, it's important to note that not all STIs cause symptoms, and some may not appear for weeks or even years after initial infection.

Specific STD Symptoms

Chlamydia

- Burning and itching at the tip of the penis and discharge in men
- Vaginal itching, discharge with odor, pain during sex, and painful urination in women

Gonorrhea

- Similar symptoms to chlamydia, including discharge and painful urination

Syphilis

- A firm, round, painless sore on the genitals or anus during the early stage
- A rash on the soles, palms, or other parts of the body, swollen glands, fever, hair loss, or fatigue in the later stages

Genital Herpes

- Occasional cold sores or "fever blisters" on the lips for HSV-1
- Fluid-filled blisters that form painful, crusted sores on the genitals, anus, thighs, or buttocks for HSV-2

Trichomoniasis

- A yellow-green discharge with a strong odor, vaginal itching, or pain during sex or urination in women

If you experience any of these symptoms or suspect you may have an STD, it's important to seek medical attention immediately for proper diagnosis and treatment.

**Sources:**

<https://www.mayoclinic.org/diseases-conditions/sexually-transmitted-diseases-stds/symptoms-causes/syc-20351240>

<https://www.healthline.com/health/sexually-transmitted-diseases>

<https://my.clevelandclinic.org/health/diseases/9138-sexually-transmitted-diseases--infections-stds--stis>

<https://www.healthline.com/health/sexually-transmitted-infections>

<https://medlineplus.gov/sexuallytransmittedinfections.html>

<https://en.wikipedia.org/wiki/Sexually_transmitted_infection>

<https://www.mayoclinic.org/diseases-conditions/sexually-transmitted-diseases-stds/in-depth/std-symptoms/art-20047081>

**Copilot prompt 1: sexually transmitted diseases symptoms**

**Copilot answer 1:**

Sexually transmitted diseases (STDs), also known as sexually transmitted infections (STIs) or venereal diseases, are infections primarily spread through sexual contact. These infections can be caused by bacteria, viruses, or parasites. Here are some common STD symptoms:

1. **Genital Discomfort**: Pain, itching, or burning in the genital area can be indicative of an STD.
2. **Abnormal Discharge**: Unusual discharge from the penis, vagina, or rectum may signal an infection.
3. **Sores or Bumps**: Open sores, blisters, or warts on the genitals, mouth, or anus could be related to an STD.
4. **Painful Urination**: Discomfort or pain while urinating may be a symptom of certain STIs.
5. **Flu-Like Symptoms**: Some STIs can cause flu-like symptoms such as fever, fatigue, and body aches.
6. **Rash or Skin Changes**: Skin rashes, redness, or other skin abnormalities may be linked to an STI.

Remember that some STDs may not show symptoms immediately, and asymptomatic carriers can still transmit the infection. [Regular testing and safe sexual practices are essential for prevention and early detection](https://www.mayoclinic.org/diseases-conditions/sexually-transmitted-diseases-stds/in-depth/std-symptoms/art-20047081)[^1^](https://www.mayoclinic.org/diseases-conditions/sexually-transmitted-diseases-stds/in-depth/std-symptoms/art-20047081)[^2^](https://www.mayoclinic.org/diseases-conditions/sexually-transmitted-diseases-stds/symptoms-causes/syc-20351240). If you suspect you have an STD, seek medical advice promptly.

**Sources:**

<https://www.mayoclinic.org/diseases-conditions/sexually-transmitted-diseases-stds/in-depth/std-symptoms/art-20047081>

<https://www.mayoclinic.org/diseases-conditions/sexually-transmitted-diseases-stds/symptoms-causes/syc-20351240>

<https://www.healthline.com/health/sexually-transmitted-diseases>
